# Introduction of dengue virus serotype 3 in the Afar Region, Ethiopia

**DOI:** 10.1080/22221751.2024.2429653

**Published:** 2024-11-18

**Authors:** Feleke Mekonnen, Bilal A. Khan, Endalkachew Nibret, Abaineh Munshea, Daniel Tsega, Demeke Endalamaw, Senait Tadesse, Gizachew Yismaw, Damtie Lankir, Jemal Ali, Mariana Ulinici, Emanuele Orsini, Urša Šušnjar, Tea Carletti, Danilo Licastro, Molalegne Bitew, Marta Giovanetti, Alessandro Marcello

**Affiliations:** a Department of Medical Laboratory sciences, College of Medicine and Health Sciences, Bahir Dar University, Bahir Dar, Ethiopia; b International Centre for Genetic Engineering and Biotechnology (ICGEB), Trieste, Italy; c Department of Virology, Dow International Medical College, Dow University of Health Sciences, Karachi, Pakistan; d Department of Biology, College of Science, Bahir Dar University, Bahir Dar, Ethiopia; e Department of Health Biotechnology, Institute of Biotechnology, Bahir Dar University, Bahir Dar, Ethiopia; f Arbovirus and influenza reference laboratory, Ethiopian Public Health Institute, Addis Ababa, Ethiopia; g Virology reference laboratory, Amhara Public Health Institute, Bahir Dar, Ethiopia; h Department of Medical Laboratory Sciences, College of Medicine and Health Sciences, Bahir Dar University, Bahir Dar, Ethiopia; i Research and Development Directorate, Amhara Public Health Institute, Bahir Dar, Ethiopia; j Department of Malaria and other vector born diseases prevention and control, Amhara Public Health Institute, Bahir Dar, Ethiopia; k Department of Diagnostics laboratory services, Afar Public Health and Research Institute, Semera, Ethiopia; l Department of Microbiology and Immunology, Nicolae Testemitanu University of Medicine and Pharmacy, Chisinau, Republic of Moldova; m Area Science Park, Trieste, Italy; n Bio and Emerging Technology Institute (BETiN), Addis Ababa, Ethiopia; o Department of Sciences and Technologies for Sustainable Development and One Health, Universita Campus Bio-Medico di Roma, Selcetta, Italy; p Oswaldo Cruz Institute, Rio de Janeiro, Brazil

**Keywords:** Ethiopia, dengue, outbreak, genomic epidemiology, Italy

## Abstract

The genetic analysis of the Dengue virus circulating in Ethiopia’s Afar region, in 2023, identified three distinct introductions with spatiotemporal clustering linked to genomes from Asia and Italy. These findings are crucial for enhancing prevention and control strategies, reinforcing the necessity to provide sustainable tools for genomic epidemiology in Africa.

## Introduction

Ethiopia has been experiencing annual dengue outbreaks since the first cases were reported in Dire Dawa in 2013 [1]. Subsequent outbreaks occurred in the Eastern and Southern districts, including Godey town and Arbaminch, in 2020 [2]. In 2019 an outbreak occurred in the Gawane District of the Afar Region in Noth-Eastern Ethiopia [3]*.* Dengue serotypes 1 and 2 (DENV-1 and DENV-2) have been reported in the country [4,5], while dengue virus serotype 3 (DENV-3) was detected only recently in the Afar Region [6]. Despite the increasing number of cases, much is still unknown about the genomic diversity and evolution of DENV lineages currently circulating in the country. To address this gap, a cross-sectional study was conducted from June to October 2023 on 384 acute febrile (AFI) patients attending 3 healthcare facilities in the Afar Region: Logia, Mille, and Gewane. The study was conducted within EXPANDIA (Expanding Diagnostics and Surveillance in Africa), a project implemented by the International Centre for Genetic Engineering and Biotechnology (ICGEB).

## The study

Patients presenting at Logia, Mille, and Gewane healthcare facilities in the Afar Region exhibiting symptoms typical of dengue were included in this study. These symptoms comprised an acute onset of high fever persisting for 2–7 days, severe joint and muscle pain, headache, retro-orbital pain, rash, nausea/vomiting, and unusual bleeding, or bruising. We included 384 patients who visited these facilities between June and October 2023. The Regional Public Health Research Ethics Review Committee, Ethiopia, approved the study (Ref. NoH/R/T/T/D/07/39). Sociodemographic and clinical information, along with other relevant data, was gathered using a structured questionnaire (Appendix 1 – Tables 1 and 2).

Malaria infection was excluded using a smear test on whole blood. Serum was obtained from venous puncture and tested on-site using the Wondfo One Step Dengue IgG/IgM test (Guangzhou Wondfo Biotech Co., Ltd., China). Serum samples that tested positive for DENV-specific IgM (32.8%, 126/384) and/or IgG (36.2%, 139/384) had to be transported at – 20°C to Addis Ababa (EPHI and BETiN) and to ICGEB for further analysis because of the lack of facilities and reagents at collecting sites to conduct NS1 antigen testing or RT PCR. Viral RNA purification from serum was performed using the QIAamp Viral RNA Mini Kit (Qiagen, Germany), and DENV RNA was detected using the Luna Universal Probe One-Step RT-qPCR Kit (NEB) using in-house pan-Dengue primers and probe. Positive samples were assigned to the DENV-3 serotype by amplifying the C-prM region, as previously described [9], followed by Sanger sequencing and data analysis using the Genome Detective DENV typing tool [8]. Samples (*n* = 7) with a cycle threshold (Ct) value of ≤32 and available epidemiological metadata – such as the date of symptom onset, date of sample collection, sex, age, municipality of residence, symptoms, and disease classification, were subjected to a nanopore whole genome sequencing using a set of tiled primers [9] following previously described protocols with slight modifications [10]. For amplification of DENV-3 genome, 30 primers were used [9] to cover the almost entire coding sequence (CDS) of the DENV-3 genome (positions 132–10139) in 800–900 bp amplicons (Appendix 2 primer list & protocol). We reconstructed phylogenetic trees to explore relationships between sequenced genomes from Ethiopia (*n* = 7) and other global regions (*n* = 351) (Appendix 3). A relatively strong correlation was observed between the sampling date and root-to-tip genetic divergence in these data sets (*r*² = 0.89, correlation coefficient = 0.94), indicating a relatively clock-like virus evolution.

**Figure 1.**
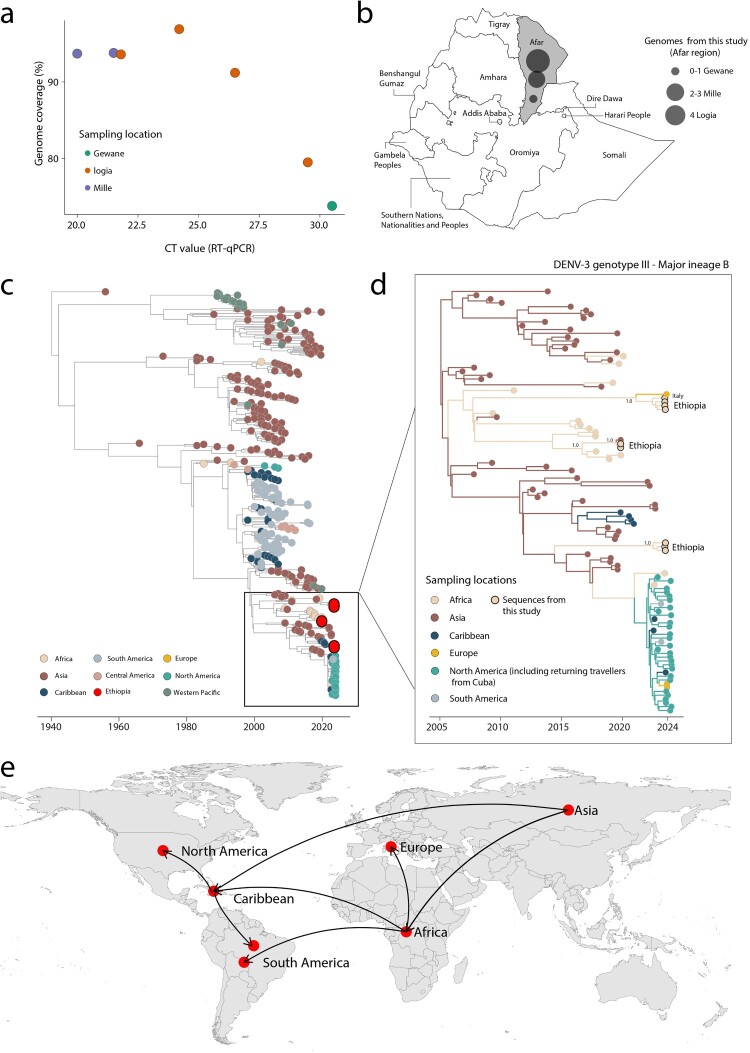
Dynamics of DENV-3 in Ethiopia. (a) The percentage of DENV genome sequenced plotted against RT – qPCR Ct value for each sample (*n* = 7). Each circle represents a sequence recovered from an infected individual in Ethiopia and is coloured by sampling location; (b) Map of Ethiopia showing the sampling locations of new DENV-3 genome sequences by region and district. The size of the circles represents the number of new genomes generated in each district; (c) Time-stamped Maximum Likelihood tree including the novel DENV-3 sequences (*n* = 7) generated in this study, along with *n* = 351 reference strains belonging to DENV-3 genotype III sampled worldwide. Tips on the tree are annotated with circles and coloured based on their locations; (d) The inset tree on the right represent a Maximum Clade Credibility (MCC) tree, which estimates divergence times, inferred using a smaller dataset (*n* = 111) which included all novel genomes from Ethiopia. Each branch is coloured according to the legend on the left; Ethiopian sequences are marked with a black circle. Support for branching structure is shown by posterior probability values at key nodes; (e) Representation of the spatiotemporal reconstruction of the spread of DENV3-III Major lineage B.

The sequencing procedure yielded an average coverage of 89%, ranging from 73.8% to 96.9% (Figure 1(a)). This allowed for the identification of the DENV-3, genotype III, major lineage B, following the recent release of the DENV classification system [11]. Genome sequences were obtained from all three districts of the Afar region: Mille, Logia, and Gewane (Figure 1(b)).

To understand the phylogenetic history of DENV-3, genotype III, we combined our recently sequenced samples (*n* = 7) from 2023 with a global set of reference samples. Our analysis revealed that the new genomes cluster together within the major lineage B and that three independent introduction events have occurred in Ethiopia over time (Figure 1(c)).

We then investigated the spatial–temporal dynamics of the Ethiopian DENV-3, genotype III, in more detail using a smaller dataset (*n* = 111) derived from the major lineage B, which has an Asian origin (Figure 1(d)). Since 2015, this lineage has spread multiple times into Africa, the Caribbean, and subsequently to North and South America, where it was recently described as a re-emerging variant circulating in the region (Figure 1(e)). Within the Ethiopian sequences, we identified three distinct clades. Clade I date back to early February 2021 (95% Highest Posterior Density – HPD: April 2019 to February 2022) and includes Ethiopian strains that cluster monophyletically with a genome sequence isolated in Rome, Italy, in 2023. Although genetic data alone cannot provide evidence of transmission direction and potential cryptic transmission must be considered due to the paucity of genomes from other locations, our analyses indicate strong clustering support for the Ethiopian sequence with the one recovered in Italy (posterior probability support = 1.0). Clade II, dating back to late June 2019 (95% HPD: January to October 2019), comprises two newly identified Ethiopian strains that cluster with a strain isolated in Asia, suggesting a potential introduction from Asia to Ethiopia. Clade III, dating back to July 2022 (95% HPD: April 2021 to October 2022), consists solely of sequences from Ethiopia and shows a clear Asian origin. Given the limited sequences from other locations and the low number of genomes from Ethiopia, this clade might represent persistent local circulation within the country. Together these findings highlight the dynamic nature of DENV-3 transmission and the complex epidemiological patterns in the country.

## Conclusions

This study underscores the dynamic and complex nature of DENV-3 transmission within Ethiopia. The introduction of at least three independent DENV-3 lineages into the Afar region, as indicated by genomic evidence, highlights a significant epidemiological pattern. These introductions have connections with distinct global regions including Asia and Europe, particularly Italy, suggesting international travel and movement as potential vectors for disease spread. Temporal and phylogenetic analyses reveal that while some of the identified strains suggest recent introductions, others indicate more established, ongoing local transmission within the country.

These findings are critical for the Ethiopian public health response, emphasizing the need for robust surveillance systems that incorporate genomic epidemiology to track virus evolution and spread effectively. Such efforts are essential not only for timely and targeted dengue control measures but also for preparing for potential outbreaks of other imported viral diseases. This study also reinforces the importance of international collaboration in genomic surveillance to better understand and mitigate the spread of infectious diseases globally. Such efforts should include the availability at community level of robust and cost-effective tools for arboviral viremic phase detection, such as NS1 antigen testing or molecular assays.

Additionally, this study produced near-complete genomes from the Afar region for the first-time using Oxford Nanopore sequencing technology. This approach could be implemented in Ethiopia and other African countries to enhance genomic surveillance and management of DENV transmission in the region.

The analysis presented in this work has limitations mainly due to scarce sampling across Ethiopia and Africa at large. More robust biogeographic and phylogeographic inferences could be made possible only by promoting further investments in genomic surveillance across the Continent. To note, a recent preprint also reported dengue sequences in Ethiopia with similar results, thus increasing the pool of data that will become available for that region (https://www.medrxiv.org/content/10.1101/2024.07.10.24310195v1).

## Supplementary Material

APPENDIX.docx

## Data Availability

The input file (e.g. alignments), and the resulting output file (log file) used and obtained in the study are shared publicly on GitHub (https://github.com/MolVir-ICGEB/DENV-Ethiopia).
