# Supplementary material for: Introduction of dengue virus serotype 3 in the Afar Region, Ethiopia
Source: Emerg Microbes Infect. 2024 Nov 18;13(1):2429653. doi: 10.1080/22221751.2024.2429653 (PMC11610227; doi:10.1080/22221751.2024.2429653)
Supplement: APPENDIX.docx [file TEMI_A_2429653_SM7737.docx]

**APPENDIX 1**

**Table 1**: Sociodemographic Characteristics of Dengue cases in Afar Region, Ethiopia. June-October, 2023.

| **Socioeconomic Status of AFI cases** | | | | |
| --- | --- | --- | --- | --- |
| **Variables** | **Dengue IgM** | | **COR (95%CI)** | **P-value** |
|  | **Positive N (%)** | **Negative N (%)** |  |  |
|  |  |  |  |  |
| **Gender** |  |  |  |  |
| Male | 102(32.4) | 213(67.6) | 1(0.643_1.928) | 0.7 |
| Female | 24(34.8) | 45(65.2) | 0 |  |
| **Age(yr)** |  |  |  |  |
| (6-15) | 3(2.4) | 7(2.7) | 0 |  |
| (16-45) | 50(39.7) | 103(39.9) | 0.85(0.214-3.388) | 0.82 |
| (>46) | 73(57.9) | 148(57.4) | 0.91(0.226-3.668) | 1 |
| **Residence** |  |  |  |  |
| Urban | 56(37.7) | 101(64.3) | 1.2(0.745-1.972) | 0.44 |
| Semi-Urban | 27(30) | 63(70) | 1.3(0.741-2.251) | 0.37 |
| Rural | 43(31.4) | 9468.6) | 0 |  |
| **Marital Status** |  |  |  |  |
| Single | 84(31.5) | 183(68.5) | 0.8(0.519-1.295) | 0.39 |
| Married | 42(35.9) | 75(64.1) | 0 |  |
| **Occupation** |  |  |  |  |
| Governmental | 3(37.5) | 5(62.5) | 0 |  |
| Pastoralist | 57(32.8) | 117(67.2) | 1.2(0.284-5.335) | 0.78 |
| Unemployed | 34(39.5) | 52(60.5) | 1.2(0.262-5.836) | 0.79 |
| House wife | 16(32.7) | 33(67.3) | 0.92(0.206-4.093) | 0.91 |
| Merchant | 3(33.3) | 6(66.7) | 1.2(0.164-8.799) | 0.86 |
| Others | 13(22.4) | 45(77.6) | 2(0.437-9.871) | 0.36 |
| Health Facility |  |  |  |  |
| Logia health center | 89(40.6) | 130(59.4) | 2.2(0.963-5.139) | 0.61 |
| Mille health center | 29(22.1) | 102(77.9) | 0.9(0.378-2.258) | 0.86 |
| Gewane health center | 8(23.5) | 26(76.5) |  |  |

| *0 = reference group, * = statistically significant (p-value < 0.05), COR (95%CI) = crudes odds ratio at 95% confidence interval* |
| --- |

**Table 2**: Clinical Characteristics of Dengue patients in Afar region, Ethiopia, June-October 2023.

| **Variables** | | **Dengue IgM** | | **COR (95%CI)** | **P-value** | | |
| --- | --- | --- | --- | --- | --- | --- | --- |
|  |  |  | |  |  |  |  |
|  | | **Positive,**  **N (%)** | **Negative N (%)** |  |  | | |
| Fever | |  |  |  |  | | |
| Yes | | 121(32.9) | 247(67.1) | 1(0.366-3.17) | 0.9 | | |
| No | | 5(31.3) | 11(68.8) |  |  | |  |
| Severe Headache | |  |  |  |  | |  |
| Yes | | 121(32.7) | 249(67.3) | 0.8(0.287- 2.67) | 0.8 | |  |
| No | | 5(35.7) | 9(64.3) |  |  | |  |
| Eye pain | |  |  |  |  | |  |
| Yes | | 62(29.5) | 148(70.5) | 21(8.145-55.770) | 0.0001 | |  |
| No | | 64(36.8) | 110(63.2) |  |  | |  |
| Muscle and Joint Pain | |  |  |  |  | |  |
| Yes | | 99(33) | 201(67) | 1(0.6199-1.744) | 0.8 | |  |
| No | | 27(32.1) | 57(67.9) |  |  | |  |
| Nausea | |  |  |  |  | |  |
| Yes | | 77(35.6) | 139(64.4) | 1.3(0.872-2.076) | 0.2 | |  |
| No | | 49(29.2) | 119(70.8) |  |  | |  |
| Swollen Gland | |  |  |  |  | |  |
| Yes | | 3(27.3) | 8(72.7) | 0.8(0.199 to 2.924) | 0.7 | |  |
| No | | 123(33) | 250(67) |  |  | |  |
| Rash | |  |  |  |  | |  |
| Yes | | 2(33.3) | 4(66.7) | 1(0.185 to 5.668) | 0.978 | |  |
| No | | 124(32.8) | 254(66.1) |  |  | |  |
| Respiratory Distress | |  |  |  |  | |  |
| Yes | | 17(37.8) | 28(62.2) | 1.3(0.673 to 2.440) | 0.45 | |  |
| No | | 109(32.2) | 230(67.8) |  |  | |  |
| Bleeding gum | |  |  |  |  | |  |
| Yes | | 2(28.6) | 5(71.4) | 0.8(0.156 to 4.266) | 0.81 | |  |
| No | | 124(32.9) | 253(67.1) |  |  | |  |
| Organ Impairment | |  |  |  |  | |  |
| Yes | | 3(37.5) | 5(62.5) | 1.2(0.290-5.248) | 0.77 | |  |
| No | | 123(32.7) | 253(67.3) |  |  | |  |
| Severe Abdominal Pain | |  |  |  |  | |  |
| Yes | | 43(36.4) | 75(63.6) | 1.3(0.801-1.994) | 0.31 | |  |
| No | | 83(31.2) | 183(68.8) |  |  | |  |
| Vomiting | |  |  |  |  | |  |
| Yes | | 50(40) | 75(60) | 1.6(1.027-2.509) | 0.038 | |  |
| No | | 76(29.3) | 183(70.7) |  |  | |  |
| Fatigue | |  |  |  |  | |  |
| Yes | | 61(31.1) | 135(68.9) | 0.9(0.558-1.310) | 0.47 | |  |
| No | | 65(34.6) | 123(65.4) |  |  | |  |
| *0 = reference group, * = statistically significant (p-value < 0.05), COR (95%CI) = crudes odds ratio at 95% confidence interval* | | | | |  |  |  |

**APPENDIX 2**

**Table 3: Primers used for DENV-3 Sequencing**

| **Primer Name** | **Sequence (5'-3')** | **Pool** |
| --- | --- | --- |
| DENV3_1_LEFT | TCAATATGCTGAAACGCGTGAGA | 1 |
| DENV3_1_RIGHT | GCCTCGGTCTTCTGAAGCTCTA | 1 |
| DENV3_2_LEFT | GGGAGTAGGAAACAGAGATTTTGTGG | 2 |
| DENV3_2_RIGHT | GAGTATTGTCCCRTGCTGCGTT | 2 |
| DENV3_3_LEFT | ACCAATAGAGGGAAAAGTGGTGC | 1 |
| DENV3_3_RIGHT | TGGCCTCGAACATCTTCCCAAT | 1 |
| DENV3_4_LEFT | GCTGAACCTCCTTTTGGGGAAA | 2 |
| DENV3_4_RIGHT | TTCCACCTCCCACACATTCCAT | 2 |
| DENV3_5_LEFT | GGCAAAAATAGTGACAGCTGAAACA | 1 |
| DENV3_5_RIGHT | TCTCCATGTTATTTGCCCTGAGAGA | 1 |
| DENV3_6_LEFT | AGGTGGACAACTTCACAATGGG | 2 |
| DENV3_6_RIGHT | CTCAGCCTCTTCCTCCCATGTT | 2 |
| DENV3_7_LEFT | GCCAGTCTTCRAGCATGAGGAA | 1 |
| DENV3_7_RIGHT | TTCCCAGGCTCTACGGCAATAA | 1 |
| DENV3_8_LEFT | AAAGACTGGAACCAAACTGGGC | 2 |
| DENV3_8_RIGHT | TGCCTGAATTCCATGAGCGTTC | 2 |
| DENV3_9_LEFT | GCAACAAAATCTGAACACACAGGA | 1 |
| DENV3_9_RIGHT | CCCTCCTCATGAGTTCCACGAA | 1 |
| DENV3_10_LEFT | AGACCATGCTCACTGGACAGAA | 2 |
| DENV3_10_RIGHT | TATGCGAGTTGGTTGTCTTGGG | 2 |
| DENV3_11_LEFT | GGAAAGACTTCAATAGGACTCATTTGTG | 1 |
| DENV3_11_RIGHT | CAGGTGATCCTTCCCAGAGTGT | 1 |
| DENV3_12_LEFT | GTGGATGGGATAATGACAATAGACCT | 2 |
| DENV3_12_RIGHT | GGTGCTCAATCACAGTTGGCAT | 2 |
| DENV3_13_LEFT | AGTGGAAGAAAGCAGAACTATAAGAGT | 1 |
| DENV3_13_RIGHT | TGGAGTTCACGTTCTCTGTCCA | 1 |
| DENV3_14_LEFT | GGAGAACCCTGGGAAGGAACAA | 2 |
| DENV3_14_RIGHT | ATGGCTGCCATTGAGGTATGTC | 2 |
| DENV3_15_LEFT | TGGACATCATATCTAGGAAAGACCAAAG | 1 |
| DENV3_15_RIGHT | AGGTTGCTCTGGAAGTGAGACC | 1 |

**PROTOCOL: Brief ONT sequencing protocol for DENV-3^1^**

Briefly 12 μl of RNA was reversely transcribed using 3 μl of Luna Script RT Super-Mix Kit (New England Biolabs, U.S.) at a thermocycler following temperature cycles of 25 °C for 2 min, 55 °C for 10 min, and 95 °C for 1 min, followed by DENV3-specific multiplex PCR in 2 pools by using Q5 Hot Start High-Fidelity 2x Master Mix (New England Biolabs, U.S.) with the cycling conditions of 98 °C for 30 seconds followed by 40 cycles of 95 °C for 15 seconds, 50 °C for 30 seconds, 72 °C for 60 seconds & final extension of 72 °C for 2 minutes. The resulting PCR products were quantified by the Qubit dsDNA High Sensitivity assay on a Qubit 4 fluorometer (Thermo Fisher Scientific, USA). The products were pooled in 1:1 ratio and purified by SPRI PCR Clean Dx beads (Aline biosciences, U.S.) which were further subjected to end-preparation by NEBNext® Ultra™ II End Repair/dA-Tailing Module (New England Biolabs, U.S.) and barcoded by Native Barcode Expansion Kit-NBD196 (Oxford Nanopore Technologies, UK). The barcoded samples were consolidated and cleaned by SPRI beads as described above followed by AMII adaptor ligation by NEBNext® Quick Ligation Module (New England Biolabs, U.S.). The final library was SPRI cleaned up, quantified and loaded into R9.4.1 flow Cell using the Ligation Sequencning Kit-LSK110 (Oxford Nanopore Technologies, UK). The sequencing data were collected for 16 hours, and the raw reads were base called and de-multiplexed in MinION Mk1C in-built tools in Minknow v 22. The resulting FASTQ files were used to generate consensus sequences by using de novo assembly in Genome Detective web tool (<https://www.genomedetective.com/>) subsequently verified it using the Epi2me alignment workflow. The resulting BAM files showed some reads exceeding the amplicon coverage areas in sequence PQ014887, which we believe may be due to nonspecific primer binding, leading to longer amplicons. However, the final sequence alignment dataset was trimmed to cover DENV3 genome positions 170–10,145 nt (9,975 nt) for all subsequent analyses.

GenBank accession numbers for the nucleotide sequence(s):
SUB14602032 DENV_Ethiopia-54 PQ014884
SUB14602032 DENV_Ethiopia-51 PQ014885
SUB14602032 DENV_Ethiopia-52 PQ014886
SUB14602032 DENV_Ethiopia-53 PQ014887
SUB14602032 DENV_Ethiopia-55 PQ014888
SUB14602032 DENV_Ethiopia-49 PQ014889
SUB14602032 DENV_Ethiopia-58 PQ014890

^1^A detailed version of this protocol is available on request.

**APPENDIX 3**

**Phylogenetic and Phylogeographic Analysis Method**

Sequence alignment was done using MAFFT and edited with AliView. A preliminary phylogenetic tree was built using IQ-TREE 2 with the GTR+G4 model (13) signal was evaluated with TempEst, and outliers were removed before creating a time-scaled tree using TreeTime. Phylogenetic relationships were inferred using a smaller dataset which included the Ethiopian strains (n=111) using BEAST v1.10.4 with a relaxed lognormal clock, skygrid population size, and GTR model. Phylogeographic analysis considered six locations: Asia, Africa, North America, Europe, South America, and the Caribbean. The analysis used an empirical distribution of 1000 trees, running the MCMC chain for 100 million iterations, sampling every 1000. Convergence for each run was assessed in Tracer v1.7.1 (ESS for all relevant model parameters >200). Maximum clade credibility (MCC) trees were summarized using TreeAnnotator v1.10.4 and visualized with the ggtree package in R. Geographic visualization was performed using SpreaD3.
